# Empyema of Pleural Cavity—Report of Four Cases*Read at Meeting Central Texas Medical Society, Waco, January 10, 1900.

**Published:** 1900-02

**Authors:** R. J. Alexander

**Affiliations:** Troy, Texas


For Texas Medical Journal.

### Empyema of Pleural Cavity—Report of Four Cases.*

R. J. ALEXANDER, M. D., TROY, TEXAS.

The four cases of empyema that are here reported possess some points of interest, however, the pathological conditions are very much the same. I do not propose giving you a lengthy paper on this subject, as all of us are familiar with the disease and its symptoms. I wish to give briefly my experience and observations from a clinical standpoint. I find less said on empyema than almost any subject in our medical literature, however, it is one of great importance, for it is a disease that demands prompt and judicious attention. The life of the patient depends upon the proper treatment. As common as the disease is, and as clear as the diagnostic symptoms ordinarily are, we sometimes find cases hard to diagnose without exploratory means.

The surgical treatment is the only treatment that we can rely upon, that is, evacuation, and free drainage.

In my hands, aspiration has availed me nothing, except as a palliative and diagnostic measure. I think as soon as we find pus cells in the effusion, we should empty the cavity and give free drainage.

---

*Read at Meeting Central Texas Medical Society, Waco, January 10, 1900.

In recent cases where we may expect the lung to react, and there is room to insert a large drainage tube between the ribs, a simple incision is all that is necessary.

I prefer the use of long tubes in all cases where they can be used; they form a siphon, and by this means a continual drainage is kept up, and the cavity can often be washed out by their use without removing dressing. After the case is sufficiently advanced it is but little trouble to cut them off and involve them in your dressing, or attach them to a piece of rubber, such as is used by a machinist for packing.

Case No. 1.—Mr. A., of Olive, Texas, a brother, aged 35. History good; had never had pneumonia, pleurisy, or la grippe. Had always been a stout, hearty man. January 22, 1896, I received a telegram to come at once. January 23, I arrived at his home, found him under the care of Dr. J. H. Reagan, a very competent physician, who had made diagnosis of abscess of liver, and in justice to him, I will say that a great many of the symptoms pointed to the correctness of his diagnosis. On inquiry, I found that patient was taken sick twenty days previous to this time, of impaction of bowel, impaction remaining several days. Immediately after receiving relief from this, he began complaining of pain in right side over the region of the liver, a continual type of fever, the temperature, so far as I was able to ascertain run from 100 to 103 degrees. I found him very much emaciated, body covered with perspiration, the skin icterous and every evidence of general sepsis.

Physical signs: Marked dyspnœa; diminished movement on right side; absence of vocal fremitus on palpation; flatness on percussion; absence of respiratory murmur on auscultation; respiration and pulse both very fast; had no cough.

I was undecided whether I was dealing with abscess of liver, or empyema, but my physical examination revealed what appeared to be an accumulation of pus in the right side. I at once recognized the fact that an operation was the only thing that promised a chance for recovery, and that under the most favorable conditions.

Had patient put on a cot in baggage car; took him to Sealy hospital, Galveston.

Called in Dr. J. E. Thompson, who, after a careful physical examination, was also unable to discriminate between empyema and abscess of liver, but aspirated in order to make positive our diagnosis. When the needle entered the pleural cavity, we found pus and withdrew 32 ounces, of a characteristic fecal order. It was now evident that he had empyema, but as so many of the symptoms indicated abscess of the liver, we thought in all probability the abscess might have ruptured into the pleural cavity, causing the empyema. Patient was prepared for operation, which was performed by Dr. Thompson, January 24, by a simple incision being made through the intercostal space between seventh and eighth ribs, about one and one-half inches long. After evacuating cavity an examination was made for an opening extending through the pleura, which we failed to find. The interspace between the ribs giving plenty of room for drainage, Dr. Thompson thought a resection unnecessary; put in two large rubber tubes and packed the opening around them with iodoform gauze, tubes extending through dressing, which was held in place by a many-tailed bandage. Long tubes were attached and the ends dropped into a vessel of water by side of bed.

Next day dressing was removed, cavity irrigated with peroxide of hydrogen and sterilized hot water.

Temperature ran about normal until the twelfth or fourteenth day, he began coughing, temperature ran up, and he passed through what appeared to be a case of bronchial pneumonia, but recovered from this in six or seven days. By 14th of February his condition was sufficiently improved for me to bring him home with me, a distance of over two hundred miles.

He got along remarkably well, appetite good, temperature continuing near normal. The irrigation of cavity with peroxide of hydrogen and sterilized water was continued once to twice a day.

March the 10th he had a return of the impaction of bowel at junction of ascending and transverse portion of colon; following the dislodgement of this, which occurred within twenty-four hours, there was some sloughing of bowel. During this time there was an elevation of temperature with increased severity of all the symptoms, but only lasted a few days. Discharge soon diminished, lung reacted, and almost obliterated the cavity. I took out one of the tubes, leaving one in for awhile, for fear of a reaccumulation of pus.

The 10th day of April he left for his home in Eastern Texas, two and one-half months after coming under my observation. In a short time he was practically well, and regained his former weight of 225 pounds, and is again strong and hearty, four years since operation.

My conclusions in this case are, that the impaction in the bowel caused the empyema. I believe there was a small fistula opening from the colon into the pleural cavity, which we failed to find in our examination. To this I attribute the fecal odor of the contents of this cavity, and in connection with this, adhesion, which in all probability caused some obstruction of the bile ducts, producing the icterus condition, and the other symptoms indicating abscess of liver. I have searched diligently for literature on the subject of empyema being produced in this way, and have failed to find a similar case recorded. It appears very rational in my mind that in an enormously distended condition of the colon, such as we often have with impaction, the colon might be pressed upward against the diaphragm where it could become adherent, and that the opposite surface of the diaphragm, from irritation, could form adhesion with the pleura. The continued pressure downward of the contents of the alimentary canal above impaction, might in this manner cause a pointing and rupture into the pleural cavity.

Case 2. Mr. C., aged 20. Had pneumonia in June, 1896. Was attacked the 26th day of June; never fully recovered from the pneumonia until the physical signs of pleurisy with effusion developed in right side. Physical examination of chest same as above case. July 15th, to confirm my diagnosis, I introduced hypodermic needle and withdrew pus. Patient was prepared for operation, which I did July 17th, by making a simple incision one inch long into the pleural cavity through the intercostal space between the seventh and eighth ribs. Irrigated cavity, put in two large rubber drainage tubes. Same dressing and treatment carried out as was in above case. October 5th lung had re-expanded, and one tube was taken out. October 15th discharge almost diminished, and was now of a serous character. At this time the other tube was taken out.

October 22d, about three months after operation, patient was considered well and discharged. It has now been over three years, and he still enjoys good health.

Case 3. Mr. M., aged 37. Family history vague. Came under my observation October 16th, 1896. About three or four weeks previous to this time, he received a knife wound in left axilla, which soon healed. Following this he gave a history of having been confined to his bed ever since he received the wound. The wound was very small, and it was thought not to enter the pleural cavity. During this time he had been treated for pneumonia, malarial fever, and intercostal neuralgia. The history of the temperature, so far as I was able to ascertain, did not show a certain crisis, but the continuance of an irregular temperature.

I found him very much emaciated, respiration about twenty-six per minute, temperature 101 in morning, 102 afternoon.

My physical examination revealed what appeared to be fluid in the pleural cavity of the left side. The apex beat of heart was on right side of sternum. I introduced hypodermic needle, and withdrew a syringe full of bloody serous fluid. October 19th I aspirated, the needle was introduced between the seventh and eighth ribs in axillary line, and withdrew about a quart of fluid. This aspiration was repeated three times.

Those aspirations seemed to have a favorable effect on his temperature and general condition for a few days, when the symptoms and physical signs would return as before. At the second aspiration the microscope revealed a few pus cells in the fluid.

I then advised an operation, realizing that more thorough drainage must be secured, but the patient's consent could not be obtained, so I again resorted to aspiration. This time withdrew a quantity of pus which showed a marked case of empyema.

On November 20th I obtained patient's consent to operate. At this time he was in a poor condition to undergo any kind of an operation. However, on November 21st I operated by making simple incision just in front of the latissimus dorsi muscle in the eighth interspace into the pleural cavity. Temperature at once came down to normal. I carried out same treatment as in above cases. He improved slowly, but by the 15th day of March he had regained strength sufficiently to walk around on the streets, and his flesh had increased until he weighed more than he ever did in life before. His lung had gradually expanded until the cavity was very small, the discharge of pus had about checked, everything pointed to a favorable recovery. On March the 20th his means became exhausted, and he returned home, very much against my will, but instructed him to have his home physician to keep up drainage and irrigation of cavity.

I have been able to obtain the following history of him since he passed out of my hands: From some cause the drainage was obstructed, and he had a reaccumulation of the pus. His home physician made a resection of a rib, from which he received no benefit except free drainage, lung failing to expand.

Some time in February, 1897, he went to Sealy Hospital, and Dr. Thompson performed Estlander's operation on him. He remained in the hospital six months, and returned home without any relief from the operation. He is now up walking around, attending to his business, with two openings into the pleural cavity, one just under the axillary space, and the other just above the tenth rib, from which discharges daily a quantity of pus.

My conclusions in this case are that empyema was caused from the knife wound entering the pleural cavity. I also think this case demonstrates the fact that the earlier thoracotomy is performed the better, and that aspiration is useless, except as a palliative and diagnostic measure.

Case 4. Mrs T., aged 20. Family history not good, three members of her family having died of a tubercular trouble. In the last part of May and June, 1899, I treated her through a case of typhoid fever. July 1st, before she had recovered from the fever, physical signs of empyema developed in left side, of which I made my diagnosis positive by use of the hypodermic syringe. She was very much emaciated and in a bad condition for an operation, but I advised an operation as the only thing that promised a shadow of a chance for recovery.

July 16th I made an incision between the seventh and eighth ribs and evacuated a quantity of pus. She held up better than I expected under this, and for a few days I had hopes of her recovery. The lung reacted, and the pus decreased in amount, but she now passed rapidly into a grave condition, failing to take nourishments, pulse from 130 to 140, and at times very irregular, temperature subnormal, involuntary evacuation from the bowel and bladder.

July 26th she died. My opinion is that the empyema in this case was of a tubercular origin.

